# PDCD4 is a CSL associated protein with a transcription repressive function in cancer associated fibroblast activation

**DOI:** 10.18632/oncotarget.11227

**Published:** 2016-08-11

**Authors:** Seung-Hee Jo, Dong Eun Kim, Andrea Clocchiatti, G. Paolo Dotto

**Affiliations:** ^1^ Cutaneous Biology Research Center, Massachusetts General Hospital, Charlestown, MA, USA; ^2^ Department of Dermatology, Harvard Medical School, Boston, MA, USA; ^3^ Department of Biochemistry, University of Lausanne, Epalinges, CH, Switzerland

**Keywords:** PDCD4, Notch/CSL signaling, transcription repression, squamous cancer, CAFs

## Abstract

The Notch/CSL pathway plays an important role in skin homeostasis and carcinogenesis. CSL, the key effector of canonical Notch signaling endowed with an intrinsic transcription repressive function, suppresses stromal fibroblast senescence and Cancer Associated Fibroblast (CAF) activation through direct down-modulation of key effector genes. Interacting proteins that participate with CSL in this context are as yet to be identified. We report here that Programmed Cell Death 4 (PDCD4), a nuclear/cytoplasmic shuttling protein with multiple functions, associates with CSL and plays a similar role in suppressing dermal fibroblast senescence and CAF activation. Like CSL, PDCD4 is down-regulated in stromal fibroblasts of premalignant skin actinic keratosis (AKs) lesions and squamous cell carcinoma (SCC). While devoid of intrinsic DNA binding capability, PDCD4 is present at CSL binding sites of CAF marker genes as well as canonical Notch/CSL targets and suppresses expression of these genes in a fibroblast-specific manner. Thus, we propose that PDCD4 is part of the CSL repressive complex involved in negative control of stromal fibroblasts conversion into CAFs.

## INTRODUCTION

Notch/CSL signaling is an important form of cell-cell communication with an established pro-differentiation and tumor suppressing function in the epidermal compartment of the skin [1]. This pathway plays also an important role in the dermal compartment. In particular, deletion or silencing of the CSL gene in dermal fibroblasts, of both mouse and human origin, results in activation of a CAF phenotype, with expression of mitogenic and pro-inflammatory cytokines and *in vivo* induction of keratinocyte tumor formation [2]. More recent work has shown that loss of CSL signaling in dermal fibroblasts results also in impaired proliferation and senescence, providing a molecular underpinning to previous findings that senescence of stromal cells and activation of a CAF phenotype are closely associated events [3].

CSL is the key mediator of canonical Notch activation at the level of transcription [4]. Under basal conditions, CSL functions as a repressor of transcription in association with co-repressors like nCor1/nCor2, SMART, histone deacetylase (HDACs) and Skip. Nuclear translocation and physical binding of the activated Notch intracellular domain (NICD) converts CSL from a transcription repressor to an activator, replacing associated co-repressors with co-activators like Mastermind-like (MAML) proteins and histone acetylases (HATs) [5]. Components of these classical CSL co-repressor and co-activator complexes have been extensively studied at both functional and ultra-structural/molecular levels [6]. Another CSL-interacting protein called RBPj-interacting and tubulin associated (RITA) has also been identified that act as a negative modulator of Notch/CSL signaling through a different mechanism, i.e. promoting CSL export from the nucleus [7]. Additional CSL-interacting proteins are likely to exist, which may play an important role in both canonical and non-canonical Notch signaling and other cell type-specific CSL functions.

PDCD4 is known as a pro-apoptotic and tumor-suppressing gene [8, 9]. Over-expression of PDCD4 inhibits tumor growth and metastasis [10], whereas PDCD4 knockout mice develop spontaneous tumors (lymphomas) [11]. Biochemically, PDCD4 can function as an inhibitor of mRNA translation of specific target genes by interacting with the eukaryotic initiation factor 4A (eIF4A) and suppressing its function [12]. In this context, mucin 1 (MUC1), an oncoprotein that is aberrantly expressed in human cancers, is a translational target of PDCD4 [13], suggesting that PDCD4 acts as a tumor suppressor by inhibiting oncoprotein translation. However, PDCD4 can also affect gene transcription. For instance, it has been reported to modulate c-Jun activity by interacting and blocking its phosphorylation, thus inhibiting AP-1 dependent transcription [14]. PDCD4 can also interact directly with the transcription factor Twist1, a key regulator of CAFs [15], interfering with its DNA binding [16]. PDCD4 mRNA is down-regulated by miR-21, and miR-21-mediated PDCD4 suppression is required for the survival of Notch-driven T-cell leukemia [17]. Of relevance for the present study, PDCD4 down-regulation by TGF-β-induced miR-21 induction was also implicated in up-regulation of the CAF marker αSMA through as yet unidentified mechanisms in differentiating myofibroblasts [18].

We report here that PDCD4 is a novel CSL interactor of functional relevance in human dermal fibroblasts (HDFs). Silencing of PDCD4 in these cells reproduces the effects of loss of CSL, leading to induction of cellular senescence and conversion into CAFs with tumor enhancing properties. While devoid of intrinsic DNA binding activity, PDCD4 is found at the promoter regions of Notch/CSL target genes and controls expression of these genes in a fibroblast-specific manner. Overall, the findings indicate that PDCD4 is part of the CSL repressive complex involved in negative control of stromal fibroblasts to CAF conversion.

## RESULTS

### Endogenous interaction of PDCD4 with CSL

**Figure 1:**
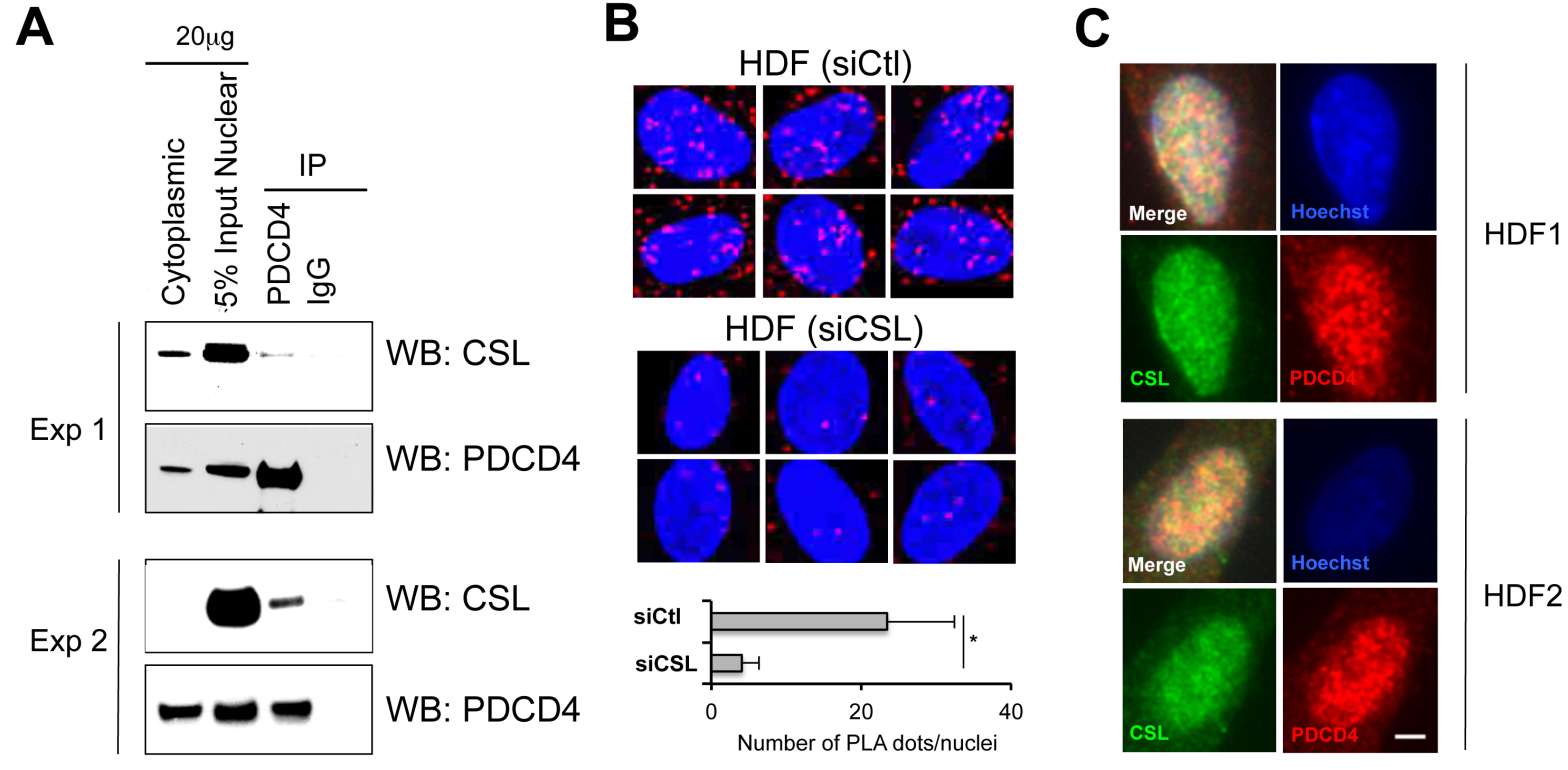
Endogenous PDCD4/CSL association and co-localization **A.** Nuclear extracts from early passage HDFs were analyzed by immune-precipitation (IP) with anti-PDCD4 antibodies or non-immune IgGs followed by immunoblotting with antibodies against CSL or PDCD4 as indicated. Shown are the results of two different experiments with HDF strains of independent origin. **B.** Proximity ligation assay (PLA) were used for *in situ* detection of CSL-PDCD4 association in HDFs. Red fluorescence foci (PLA signals) represent the interaction between CSL and PDCD4, and were analyzed by confocal microscopy with DAPI staining of nuclei (blue). The specificity of CSL-PDCD4 PLA signals was confirmed by the significant reduction of PLA signal in HDFs with siRNA-mediated CSL gene silencing. Shown are representative PLA images and the average number of dots per nucleus in HDFs plus/minus siRNA-mediated CSL gene silencing. PLA dots were counted from at least 30 cells in four fields. *p < 0.001, two-tailed *t*-test. **C.** Double immunofluorescence analysis of HDFs with anti-CSL (green) and anti-PDCD4 (red) antibodies and DAPI for nuclear DNA staining (blue). Images are representative of three independent experiments with two different strains of HDFs. Bar, 10μm.

Screening of a yeast two-hybrid cDNA library with a CSL bait pointed to PDCD4 as a potential interactor. No direct binding was detected after over-expression of the two proteins in an exogenous system. However, a weak but consistent association between endogenous PDCD4 and CSL was found by co-immunoprecipitation assays with nuclear extracts derived from HDFs (Figure 1A). For independent confirmation, we performed proximity ligation assays (PLA) for detection and localization of protein-protein interactions. Positive punctate signals resulting from the close juxtaposition of the antibodies were found in HDFs, with > 6 folds reduction in HDFs with CSL gene silencing as control of specificity (Figure 1B). Nuclear co-localization of the two proteins was also observed by double immunofluorescence analysis of HDFs (Figure 1C).

### PDCD4 gene silencing reproduces the effects of CSL loss in induction of CAF markers and Notch target genes

**Figure 2:**
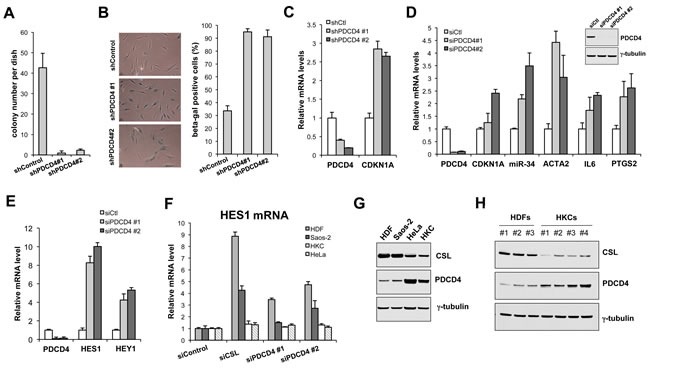
PDCD4 as negative regulator of dermal fibroblast senescence and CAF activation **A.** Clonogenecity assays. HDFs were infected with two shRNA PDCD4-silencing lentiviruses in parallel with empty vector control, followed by selection with puromycin for 48hrs, then plated under low density conditions on triplicated dishes (1000 cells per 60mm dish). Colony formation was measured by crystal violet staining after 14 days. **B.** Senescence Associated β-galactosidase activity (SA β-gal) assay. HDFs infected with the PDCD4-silencing and control lentiviruses as in the previous panel were stained with SA β-gal at 7 days after infection. Representative images (left panel) and quantification of positive SA β-gal staining in ∼100 cells (right panel) are shown. **C.** Parallel cultures of cells as in the previous panel were analyzed for expression of PDCD4 gene silencing and expression of the senescence-determinant CDKN1A gene by RT-PCR. **D.**, **E.** HDFs were transfected with two different PDCD4-silencing siRNAs in parallel with scrambled siRNA control, followed, 72hrs after transfection, by analysis of expression of the indicated genes by real time RT-PCR. Insert: Immunoblot analysis of HDFs plus/minus siRNA-mediated silencing of PDCD4. **F.** HDFs, Saos-2 cells, human primary keratinocytes (HKCs) and HeLa cells were transfected with siRNAs for silencing of the CSL or PDCD4 genes in parallel with scrambled siRNA controls, followed by determination of HES1 expression by real time RT-PCR. **G.**, **H.** Immunoblot analysis of CSL and PDCD4 expression in several HDF and HKC strains and Saos-2 and HeLa cells as indicated, with tubulin as equal loading control.

CSL gene silencing in HDFs leads to premature senescence together with acquisition of a CAF phenotype [3]. Similarly, PDCD4 gene silencing triggered cellular senescence, as assessed by reduced clonogenecity (Figure 2A) and increased Senescence Associate β-galactosidase (SA β-gal) (Figure 2B), and resulted in the concomitant induction of senescence- and CAF-determinant genes (Figure 2C and 2D).

In parallel with the increase of senescence- and CAF-determinant gene expression, down-modulation of PDCD4 expression in HDFs resulted in induction of the “canonical” Notch/CSL targets HES1 and HEY1 (Figure 2E), indicating that PDCD4 functions also as a negative co-regulator of these genes. Interestingly, up-regulation of these genes by both CSL and PDCD4 gene silencing was observed in HDFs and osteosarcoma (fibroblast-like) cell line Saos-2 cells, but not human primary keratinocytes (HKCs) or HeLa cells (Figure 2F). Only genes to which CSL is constitutively bound are induced when CSL levels decrease and differential binding of CSL to target genes can depend, among other things, on its expression levels, which can vary among cell types. Consistent with this possibility, immunoblot analysis showed consistently higher levels of CSL expression in multiple HDF strains and Saos-2 cells than in HKCs and HeLa cells, with an opposite pattern of PDCD4 expression (Figure 2G and 2H).

### PDCD4 is a part of the CSL repressive complex in controlling gene transcription

**Figure 3:**
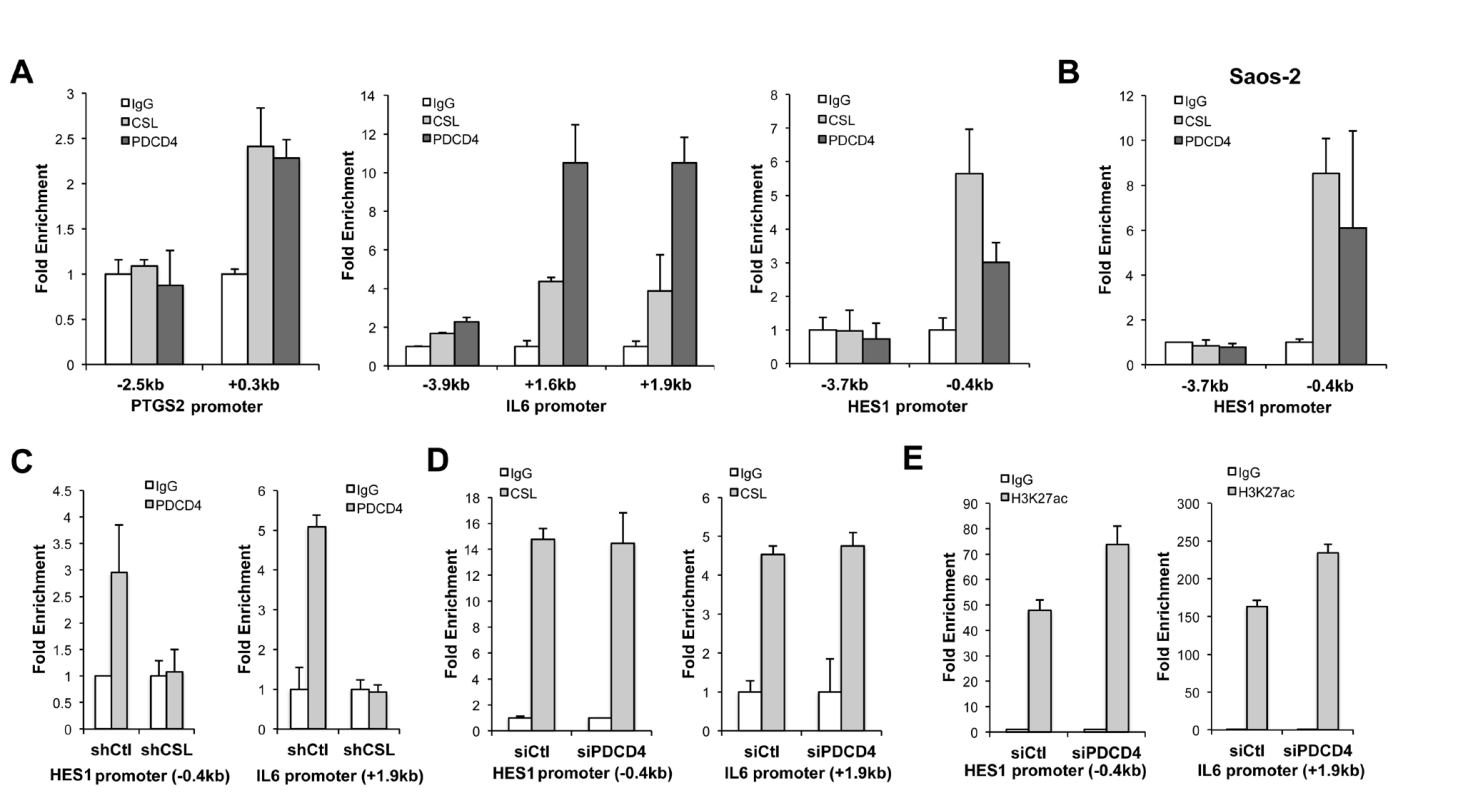
Convergent binding of PDCD4 and CSL to common target genes **A.** HDFs were assayed by ChIP with anti-CSL and PDCD4 antibodies in parallel with non-immune IgGs followed by real-time PCR of the indicated promoter regions of the PTGS2, IL6, and HES1 gene to which CSL was previously shown to bind [3]. Similar results were obtained in a second independent experiment (Supplementary Figure 1). **B.** Similar ChIP assays were performed with Saos-2 cells followed by PCR amplification of the indicated regions of the HES1 promoter. **C.** HDFs plus/minus shRNA-mediated CSL gene silencing were analyzed by ChIP with anti-PDCD4 antibodies in parallel with non-immune IgGs, followed by PCR of the indicated promoter regions of the HES1 and IL6 genes. **D.**, **E.** HDFs plus/minus siRNA-mediated PDCD4 gene silencing were analyzed, 72hrs after siRNA transfection, by ChIP with anti-CSL (D) or anti-H3K27Ac (E) antibodies in parallel with non-immune IgGs followed by PCR of the indicated promoter regions of the HES1 and IL6 genes.

CSL has an intrinsic transcription repressive function and the expression of target genes to which it binds with high affinity is induced when levels of CSL decrease [19, 20]. PDCD4 is devoid of intrinsic DNA binding activity. However, an attractive possibility is that PDCD4 may associate with CSL at specific target genes and affect their expression. Chromatin immunoprecipitation (ChIP) assay showed the binding of PDCD4 to the promoter regions of CAF genes, such as PTGS2 and IL6, at the same sites as CSL binds and also to the promoter region of canonical Notch target HES1 (Figure 3A). PDCD4 association to CSL binding region of HES1 was also found in Saos-2 cells (Figure 3B). Importantly, we found that PDCD4 binding to these genes was lost in CSL-silenced HDFs, indicating that it is CSL-dependent (Figure 3C).

To further probe into the function of PDCD4, we assessed the consequences of its down-modulation on binding of CSL to its target genes. PDCD4 silencing did not change the binding of CSL to the promoter regions of IL6 and HES1 (Figure 3D), but resulted in increased levels of H3K27ac, a mark of activated chromatin (Figure 3E), consistent with PDCD4 being part of the CSL transcription repressive complex.

### *In vivo* role of PDCD4 in CAF activation

**Figure 4:**
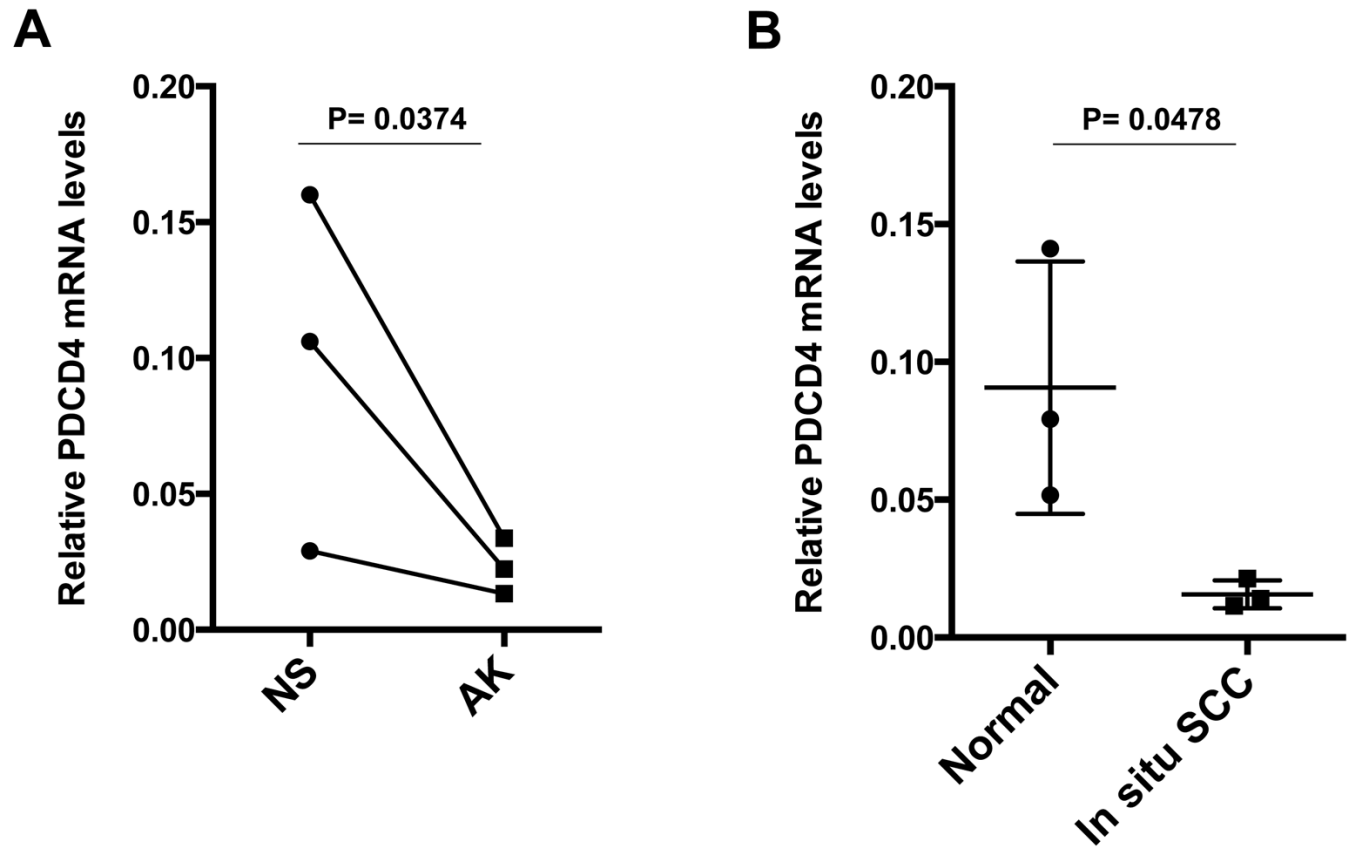
PDCD4 expression is down-regulated in stromal fibroblasts of premalignant and malignant keratinocitic lesions **A.** Laser Capture Microdissection (LCM) and RT-qPCR analysis of PDCD4 expression in stromal cells underlying actinic keratosis (AK) and surrounding normal skin (NS) from the same patients, using β-actin normalization. The same samples were previously analyzed for CSL expression, ruling out leukocyte and endothelial cell contamination by RT-qPCR of corresponding markers [3]. *n* = 3, Two-tailed paired *t*-test. **B.** LCM and RT-PCR analysis of PDCD4 expression in stromal fibroblasts, identified by PDGFRα immunofluorescence, of *in situ* SCC lesions *versus* normal skin from other individuals; mean +/-s.e.m. two-tailed unpaired *t*-test.

To assess whether the above findings are of *in vivo* significance, we started by assessing the level of PDCD4 expression in stromal fibroblasts in premalignant or malignant lesions. Laser capture microdissection (LCM) analysis showed that PDCD4 was down-regulated in stromal fibroblasts underlying premalignant skin actinic keratosis (AKs) lesions relative to fibroblasts of adjacent unaffected skin (Figure 4A), and in stromal cells of *in situ* SCC relative to those of normal individuals (Figure 4B).

**Figure 5:**
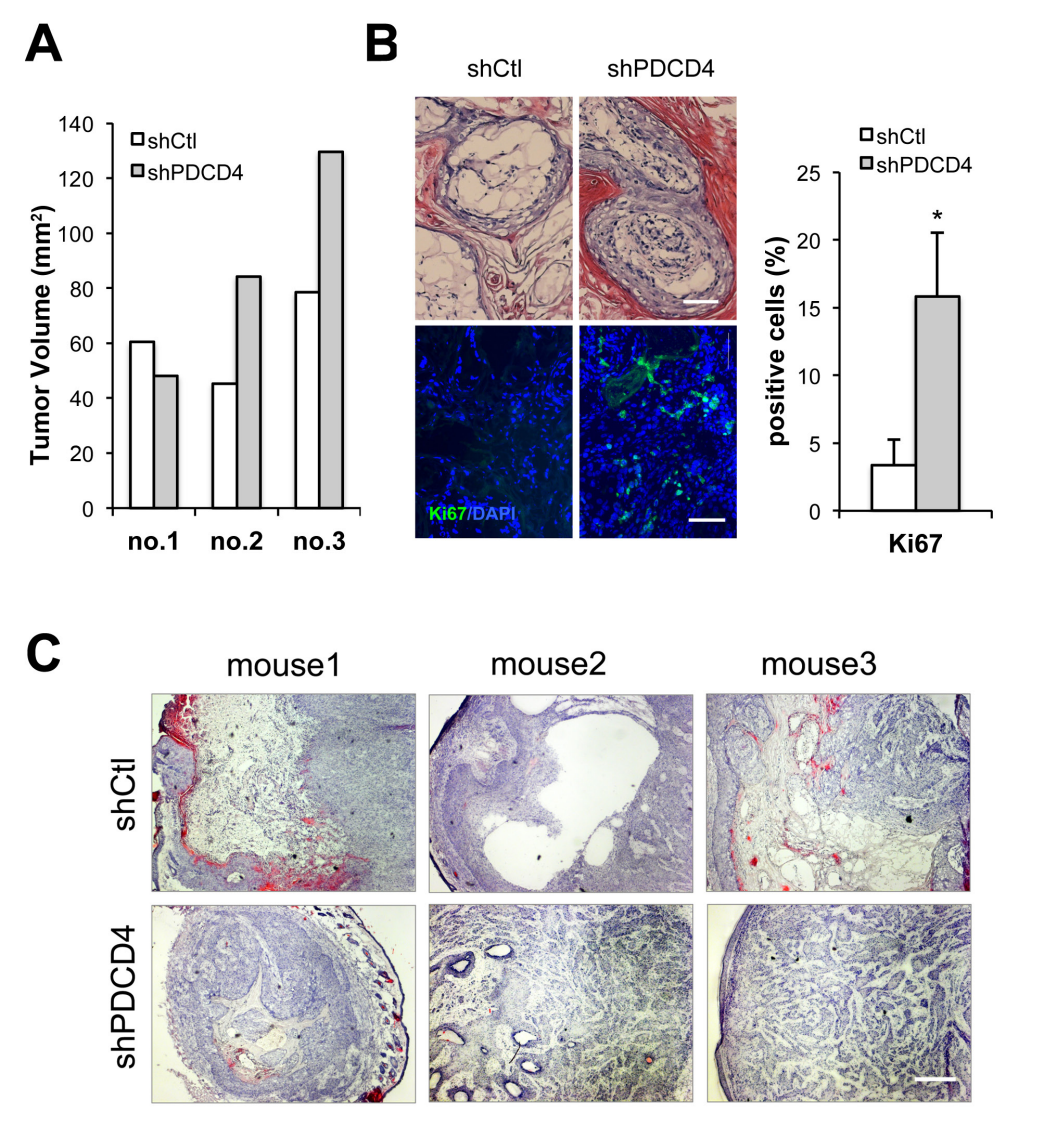
PDCD4 depleted fibroblasts enhance tumorigenic behavior of keratinocyte-derived SCC cells **A.** Weakly tumorigenic SCC13 cells were admixed with dermal fibroblasts freshly infected with a PDCD4 gene silencing lentivirus *versus* empty vector control (as in Figure 1) followed by intradermal injections into NOD/SCID mice. Mice were sacrificed at 3 weeks after injection and tumor volume was calculated using the formula V = (*X*^2^ x *Y)/2*. (X is the width and Y is the length of the tumor.) **B.** Tumors from the previous experiment were processed for histological examination and H&E staining (top panel) and immunofluorescence with antibody against the Ki67 proliferation marker with DAPI for nuclear staining (lower panel). Shown are representative images as well as a quantification of Ki67 positive nuclei using Image J. * *p*< 0.05, two-tailed t-test, 100μm. **C.** SCC13 cells were admixed with HDFs plus/minus PDCD4 gene silencing, as in the previous experiment, followed by parallel injections into mouse ears. At 3 weeks after injection, mice were sacrificed and ear lesions were processed for H&E staining. Bar, 400μm.

**Figure 6:**
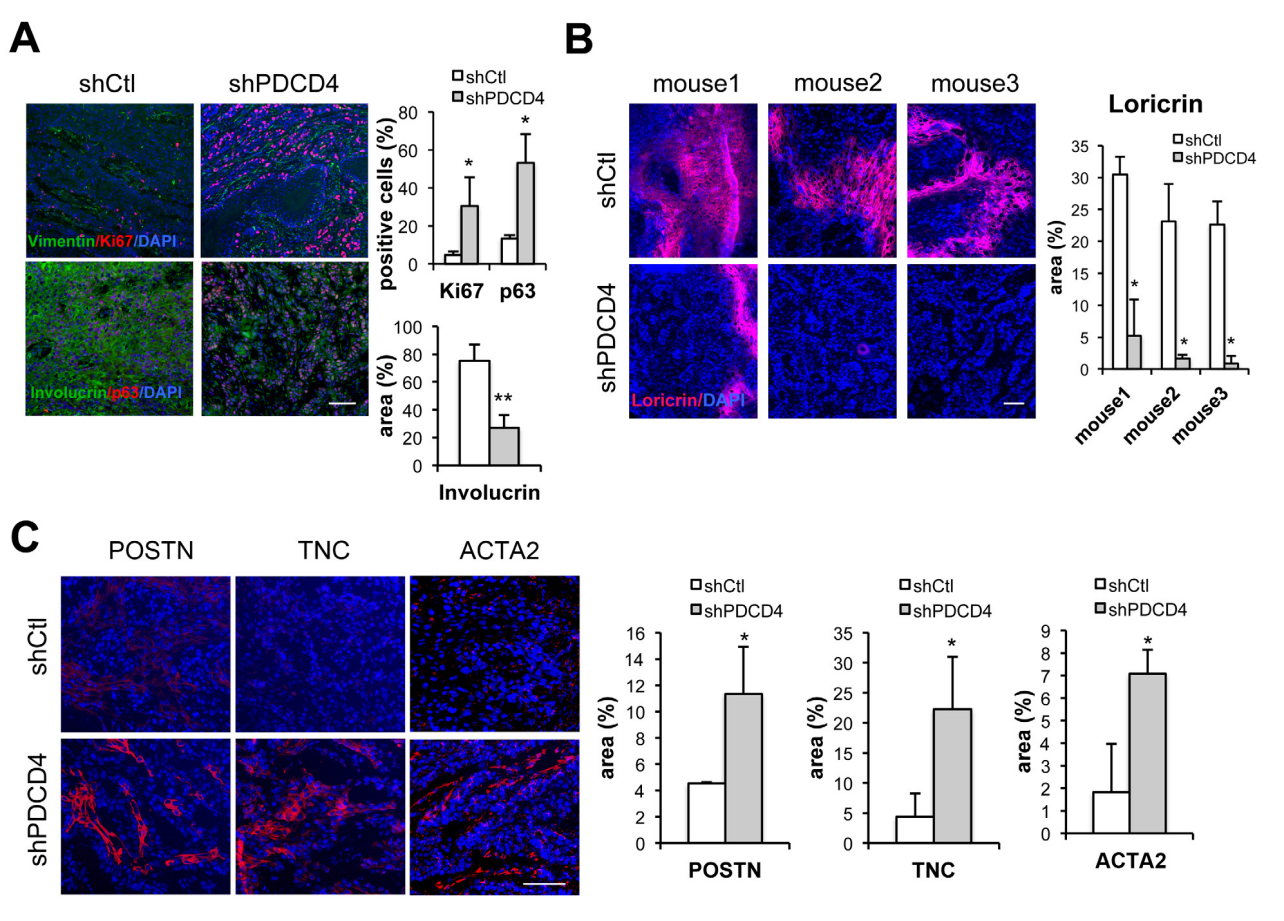
Dermal fibroblasts with PDCD4 gene silencing promote formation of SCC with higher proliferative index and suppressed differentiation **A.** Immunofluorescence of ear lesions formed by SCC13 cells with HDFs plus/minus shRNA-mediated silencing of PDCD4 with antibodies against Ki67 and Vimentin (upper panels) or Involucrin and p63 (lower panels). Shown are representative images as well as a quantification of Ki67, p63, and involucrin staining. Percentages of Ki67 positive nuclei in Vimentin negative SCC cells were quantified by Adobe Photoshop software using Lasso tool in three different fields from 3 ear pairs. p63 positive nuclei over total nuclei were quantified using Image J. * < 0.05, ** < 0.005, two-tailed *t*-test. Bar, 100μm. **B.** Immunofluorescence analysis of ear lesions formed by SCC13 cells with HDFs plus/minus shRNA-mediated silencing of PDCD4 with antibodies against Loricrin with DAPI for nuclear staining. Positive areas relative to entire lesion size (expressed as %) were quantified using Image J software. * < 0.05, two-tailed *t*-test, Bar, 100μm. **C.** Immunofluorescence analysis of ear lesions formed by SCC13 cells with HDFs plus/minus shRNA-mediated silencing of PDCD4 with antibodies against Periostin (POSTN), Tenascin C (TNC), and α-Smooth muscle actin (ACTA2)with DAPI for nuclear staining. Shown are representative images as well as quantification of positive areas relative to entire lesion size (expressed as %) in 3 different fields from 3 ear pairs using Image J software. * < 0.05, two-tailed *t*-test, Bar, 100μm.

A key feature of CAFs is their ability to enhance proliferation of neighboring cancer cells. Accordingly, HDFs plus/minus PDCD4 gene silencing were tested by intradermal tumorigenicity assays with a keratinocyte-derived SCC cell line (SCC13). Tumor growth was enhanced when SCC13 cells were injected into mouse back skin with HDFs with PDCD4 gene silencing (Figure 5A). Tumors formed in the presence of HDFs with PDCD4 knockdown had higher cellularity and Ki67 proliferative index than with controls (Figure 5B). The same combination of cells was tested by alternative tumorigenicity assay based on injection into mouse ears. Histological analysis revealed that SCC13 cells forms tumors with higher cellularity when admixed with HDFs with PDCD4 knockdown, whereas tumors formed with control HDFs showed large empty or necrotic areas associated with the terminal differentiation cornification process (Figure 5C). In addition, greater positivity of the Ki67 proliferative index as well as the p63 proliferative marker was found in lesions formed with HDFs with PDCD4 silencing, while expression of the involucrin and loricrin differentiation marker was less (Figure 6A, 6B and Supplementary Figure 2A), showing tumors formed with HDFs with PDCD4 silencing contain more undifferentiated tumor cells. Furthermore, expression of key CAF markers, such as αSMA, periostin, and tenascin C, was increased in tumors with HDFs with PDCD4 silencing (Figure 6C and Supplementary Figure 2B).

Thus, PDCD4 exerts a significant *in vivo* function in suppression of CAF activation, which parallels that of CSL.

## DISCUSSION

The CSL protein functions as a transcription regulator in concert with various co-repressors or co-activators [5]. Besides the CSL associated proteins involved in canonical Notch-dependent control of transcription, there are others with distinct or separate functions. For instance, the CSL-interacting protein DDX5 plays a more peripheral role enhancing formation of the CSL co-activator complex [21, 22], while the pancreas-specific transcription factor 1 (Ptf1) interacts and functions together with CSL independently of Notch signaling [23]. The present findings indicate that PDCD4 is also connected with CSL function, in a cell type specific manner impinging on negative control of stromal fibroblasts to CAF activation. Interestingly, no direct binding was detected after over-expression of the two proteins in a heterologous system, which suggests that their interaction requires other intermediate proteins. However, the molecular mechanism by which PDCD4 controls CSL repressive complex formation needs further investigation. SIRT1 and LSD1 proteins were recently found as a co-repressor component of CSL/Notch target genes with histone modifying activity [24] and it will be very interesting to assess whether PDCD4 is part of this new complex.

Cancer-associated fibroblasts (CAFs) are well known to support tumor growth by secreting growth factors/cytokines [25, 26] and also by providing energy-rich metabolites [27]. Many signaling pathways and transcription factors have been implicated in control of CAF gene expression [28], However, surprisingly little is known of the transcriptional changes involved in the early transition of normal fibroblasts into CAFs. Stromal alterations associated with cellular senescence and tissue aging can play an important role in the early stages of epithelial cancer development, with subsequent changes leading to cancer/stromal cell expansion [29]. Compromised Notch/CSL function is key at these early stages, leading to the concomitant induction of stromal fibroblast senescence and production of the “senescence messanging secretome” (SMS), a battery of tumor promoting cytokines, growth factors and matrix remodeling proteins that are a trademark of fully established CAFs [3]. Concurring with these findings, CSL is less expressed in stromal cells adjacent to skin premalignant lesions like actinic keratosis (AK) and *in situ* SCC relative to those of unaffected skin [3]. In parallel with CSL, we have shown here that PDCD4 expression is also decreased in stromal fibroblasts of these early lesions. Like with CSL, we have found that silencing of PDCD4 was sufficient to induced fibroblast senescence and SMS production and, importantly, enhance squamous cell tumor formation. While PDCD4 has been extensively studied in cancer cells and tissues [9], there are very limited studies on PDCD4 functions in stromal cells, specifically one implicating it in negative regulation of αSMA through unspecified mechanisms [18]. Besides associating with CSL, we have shown here that PDCD4 controls transcription of CAF effector genes as well as canonical Notch target genes in a fibroblast-specific manner with potentially important implications also for other systems in which stromal alterations are involved in control of epithelial cancer.

## MATERIALS AND METHODS

### Cells and viruses

HDFs and HKCs were obtained and cultured as previously described [2, 30]. Human skin samples for primary cell preparation were obtained from abdominoplasty procedures at Massachusetts General Hospital (MGH 2008-P-001742/2, Boston, Massachusetts, USA). Saos-2 and HeLa cell lines were purchased from the American Type Culture Collection (ATCC) and cultured in DMEM with 10% FBS. Human skin SCC13 cells were originally reported in [31] and cultured in serum-free keratinocyte SFM medium.

The lentiviral vectors encoding shRNA against PDCD4 (TRCN0000059081 and TRCN0000059078) were purchased from Openbiosystem. Lentiviruses were produced by cotransfection of HEK293 cells (Lipofectamine 2000, Invitrogen) with pLKO.1-shPDCD4 and lentiviral package plasmids.

### Co-immunoprecipitations and western blotting

Nuclear extracts were prepared using NE-PER nuclear and cytoplasmic extraction kit (Thermo Scientific) according to manufacturer's instruction. 200 μg of nuclear fraction were incubated overnight at 4°C with PDCD4 antibodies (Cell Signaling Technology) or control non-immune IgGs. Dynabeads Protein G were incubated with the antigen-antibody complex for 4 hours at 4°C. Beads were washed three times with NP-40 buffer (20 mM Tris-HCl buffer, pH 8 containing 150 mM NaCl, 1 % NP-40, 1 % glycerol, and 2mM EDTA) and eluted in 50 ul of 2x SDS sample buffer at 95°C for 5 minutes and analysed by gel electrophoresis and immunoblotting.

For western blotting, cells were lysed using RIPA buffer (50mM Tris-HCl (pH 7.5), 150mM NaCl, 1% NP-40, 0.5% sodium deoxycholate, 1mM EDTA) with protease inhibitor cocktail (Roche). Proteins were separated on NuPAGE 4-12 % Bis-Tris gels (Invitrogen) and transferred to a nitrocellulose membrane (Invitrogen). Antibodies specific to PDCD4 (Cell Signaling Technology), CSL (Cell Signaling Technology), and γ-tubulin (Sigma) were commercially obtained.

### Immunofluorescence and proximity ligation assay

Cells were fixed with 4% formaldehyde and permeabilized in 0.1% Triton X-100. After blocking with 5% donkey serum in PBS, cells were incubated with primary antibody solution containing mouse monoclonal PDCD4 antibody (Santa Cruz Biotechnology) and rabbit monoclonal CSL antibody (Cell Signaling Technology). After washing, cells were incubated with goat anti-rabbit Alexa Fluor 488 and donkey anti-mouse Alexa Fluor 598 antibodies (Invitrogen), then mounted with Vectashield mounting medium with DAPI (Vector Laboratories).

Proximity ligation assay was performed using Duolink PLA kit (Sigma) according to manufacturer's protocol. Briefly, cells were fixed with 4% formaldehyde and permeabilized in 0.1% Triton X-100. After blocking with PLA blocking solution, cells were incubated with primary antibody solution containing PDCD4 and CSL antibodies. After washing with PLA wash buffer, cells were incubated with PLA probes, anti-rabbit PLUS, anti-mouse MINUS, then washed, ligated, amplified by rolling circle amplification. Images were obtained with a Nikon Eclipse Ti confocal microscope.

### Senescence assay and clonogenicity

For clonogenicity assay, 1000 cells were plated in 60mm dishes in triplicate and cultured for 14 days. The colonies were fixed with 70% ethanol and stained with 1% crystal violet.

Senescence β-galactosidase (SA-β-Gal) activity assays was performed using senescence-galactosidase staining kit (Cell Signaling Technology) according to manufacturer's protocol. For each strain, a minimum of 100 cells was counted.

### siRNA transfection and realtime RT-PCR analysis

HDFs (4 × 10^5^ cells) were plated in 60mm dishes 24 hours prior to transfection and transfected with siRNA using Hiperfect (Qiagen). After 72 hours of transfection, cells were removed and total RNAs were isolated using RNeasy mini kit (Qiagen) according to the manufacturer's protocol. RNAs were reverse-transcribed into cDNA using the iScript^™^ cDNA synthesis kit (Bio-Rad), then analysed in triplicate with gene-specific primers and 36β4 normalizaion. The primers used for realtime PCR analysis are listed in Supplementary Table 1. siRNAs for PDCD4 (Stealth RNAi HSS120545 and HSS120546) and CSL (Silencer Select siRNA s7252) were purchased from Invitrogen and Ambion.

### Intradermal tumorigenicity assay

SCC13 cells (1 × 10^6^ cells) were admixed with HDFs (5 × 10^5^ cells) with shRNA-mediated silencing of PDCD4 or control in 150 μl of growth factor-reduced matrigel (BD Bioscience) and intra-dermally injected into the back skin of 6-week-old NOD/SCID mice (Taconic Farms Inc.) as previously described [30]. For the ear injection, SCC13 cells (1 × 10^5^ cells) were admixed with equal numbers of HDFs with shRNA-mediated silencing of PDCD4 or control. Cells were resuspended in 3 μl of Hanks’ balanced salt solution and then injected intradermally into the left and right tip of the ear dermis of 10-week-old NOD/SCID mice using a Hamilton microsyringe fitted with 33 gauge needle as performed in [3]. Mice were sacrificed 3 weeks after injection and tumors were removed for analysis.

Tissue immunofluorescence was performed as before [2, 3]. Anti- ki67 antibody, anti-Vimentin antibody (Abcam), anti-p63 antibody (Santa Cruz Biotechnology), anti-Involucrin antibody (Sigma), anti-Periostin antibody (Abcam), anti-Tenascin C antibody (Santa Cruz Biotechnology), and anti-αSMA antibody (Santa Cruz Biotechnology) were used. Quantification of all images of tissue immunofluorescence staining was performed using ImageJ and Adobe Photoshop software.

All animal studies were performed following the approved Institutional animal protocol procedure (IACUC-MGH).

### Laser capture microdissection (LCM)

LCM was performed as described previously [3]. Briefly, laser-captured cells from paraffin sections were collected using an Arcturus XT microdissection system (Applied Biosystems) and RNAs were purified by RNeasy FFEP kit (Qiagen). For immunofluorescence-guided LCM, frozen blocks of normal skin and in situ SCCs were freshly cut, immediately fixed and blocked. Sections were incubated with a mixture of fluorescein isothiocyanate (FITC)-conjugated antibodies against PDGFRα (Santa Cruz Biotechnology) and propidium iodide. The air-dried sections were then used for fluorescence-guided LCM using an Arcturus XT microdissection system as before. RNA samples were analysed in triplicate with gene-specific primers.

### Chromatin immunoprecipitation (ChIP)

ChIP assays were performed as previously described [3]. Briefly, cells were cross-linked with 1% formaldehyde and chromatin fragmentation was done by sonication using a Bioruptor (Cosmo-Bio Diagenode). Fragmented chromatins were diluted 10-fold with ChIP dilution buffer (0.01% SDS, 1.1% Triton X-100, 2mM EDTA, 55mM Tris-HCl, pH 7.9, 167mM NaCl) and immunoprecipitated with PDCD4 antibody, CSL antibody or H3K27Ac antibody (Cell Signaling Technology). The primers used for realtime PCR analysis are listed in Supplementary Table 1.

### Statistics

Data are presented as mean ± standard deviation (s.d.), mean ± standard error of the mean (s.e.m.). All realtime RT-PCR samples were tested in triplicate, and statistical significance of the results was assessed by two-tailed unpaired or paired *t*-test. *P*-value of < 0.05 was considered statistically significant.

## SUPPLEMENTARY MATERIAL TABLE AND FIGURES


