# Supplementary material for: PDCD4 is a CSL associated protein with a transcription repressive function in cancer associated fibroblast activation
Source: Oncotarget. 2016 Aug 11;7(37):58717–27. doi: 10.18632/oncotarget.11227 (PMC5312270; doi:10.18632/oncotarget.11227)
Supplement: Supplementary file 1 [file oncotarget-07-58717-s001.pdf]

PDCD4 is a CSL associated protein with a transcription repressive function in cancer associated fibroblast activation

Supplementary Material

Supplemental Table 1. DNA oligonucleotide primers used in the study.

GENE	Forward	Reverse
**Primers for RT-PCR**		
PDCD4	GGGCAGCAATCTGTCAATCA	TCCTTAAGGCAATGTTCAGCTTC
CSL	CAAAAGTTGCACAGAAGTCATA	TGCTGCATTTCTTGGTCAC
CDKN1A	GATTAGCAGCGGAACAAGGA	CAACTACTCCCAGCCCCATA
miR-34	CCTCCAAGCCAGCTCAGTTG	TGACTTTGGTCCAATTCCTGTTG
ACTA2	AGCGCAAATACTCTGTCTGG	AGGCATAATTCCACAGGACA
IL6	GTGTGAAAGCAGCAAAGAGGCACTG	TGCCTTTTTCTGCAGGAACTGGATC
PTGS2	ACCCTCTATCACTGGCATCCCCTTCT	TGCCTGCTCTGGTCAATGGAAGC
HES1	GGTGCTGATAACAGCGGAAT	TGAGCAAGTGCTGAGGGTTT
HEY1	CTGTGGTTGCCAAGGTATTT	CAACAGAGGTCAAACCCAGT
36B4	GCAATGTTGCCAGTGTCTGT	GCCTTGACCTTTTCAGCAAG
**Primers for ChIP**		
PTGS2 -2.5kb	CAGCAGAAGGGGGCAGTAAA	TTCAATGTTTCTGTGTCTATTTTAGGC
PTGS2 0.3kb	CCTGGGTCTATCCCAGTACTCC	TAATGATCAGTGCTTGTGGGAAA
IL6 -3.9kb	AGTGGCAATGTGGGAGAG	TGCCCTGCCCTCTCCCAA
IL6 +1.6kb	ACTGTCAAATGTTTAAAACTCCC	GCCAGGGGCAGCCAGAGA
IL6 +1.9kb	CACACCACATGTCCCTAAA	ACAGCTTCTTCTGGGGTTGAGTCC
HES1 -3.7kb	CCACACAGGAAAACCCTACG	TGCCCTGTCATGTTCTGAAG
HES1 -0.4kb	CTCCTCCCATTGGCTGAAAG	GCTGTTCCAGGACCAAGGAG

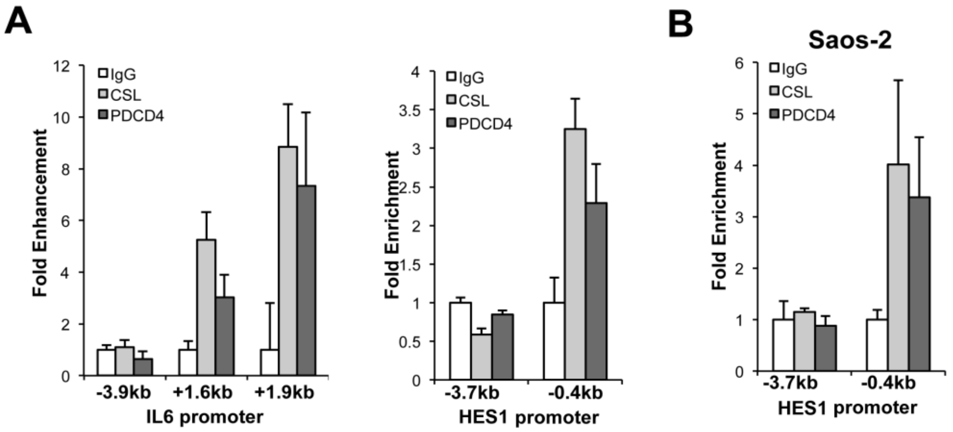

Supplementary Figure 1. Convergent binding of PDCD4 and CSL to common target genes.

(A) HDFs and (B) Saos-2 cells were assayed by ChIP with anti-CSL and PDCD4 antibodies in parallel with non-immune IgGs followed by real-time PCR of the promoter regions of the IL6 and HES1 gene.

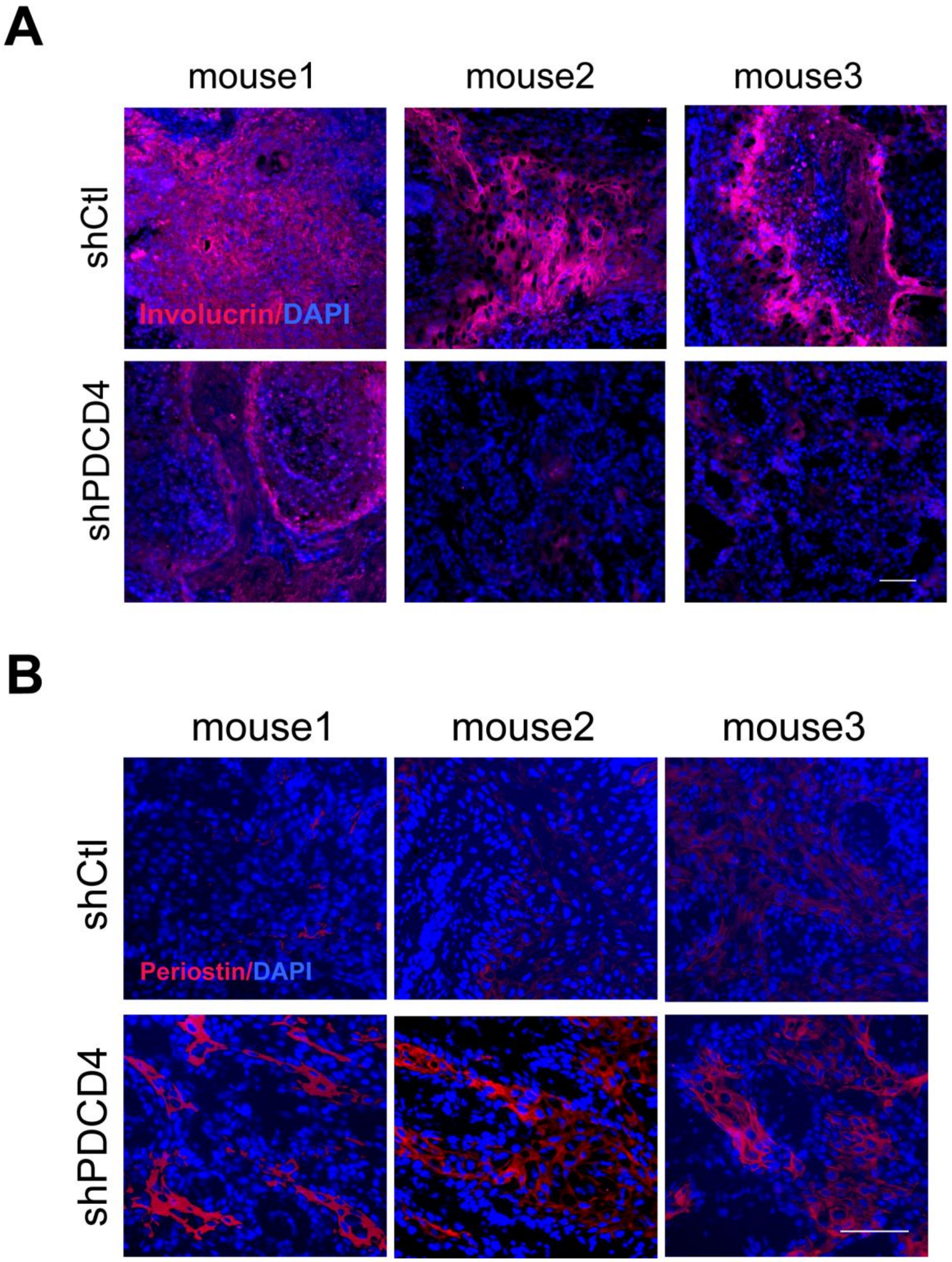

Supplementary Figure 2. Dermal fibroblasts with PDCD4 gene silencing enhance tumorigenic behavior of keratinocyte-derived SCC.

SCC13 cells were admixed with HDFs freshly infected with a PDCD4 gene silencing lentivirus versus empty vector control followed by parallel injections into mouse ears. At 3 weeks after injection, mice were sacrificed and ear lesions were stained with antibodies against Involucrin (A) and Periostin (B). Shown are representative images of staining. Bar, 100μm.
